# Mental health care utilization among men with castration‐resistant prostate cancer receiving abiraterone or enzalutamide

**DOI:** 10.1002/cam4.6237

**Published:** 2023-06-16

**Authors:** Phoebe A. Tsao, Jennifer Burns, Kyle Kumbier, Jordan B. Sparks, Shami Entenman, Lindsey E. Bloor, Amy S. B. Bohnert, Ted A. Skolarus, Megan E. V. Caram

**Affiliations:** ^1^ Division of Hematology/Oncology, Department of Internal Medicine University of Michigan Medical School Ann Arbor Michigan USA; ^2^ Veterans Affairs Health Services Research & Development, Center for Clinical Management and Research Veterans Affairs Ann Arbor Healthcare System Ann Arbor Michigan USA; ^3^ Institute of Health Policy and Innovation, University of Michigan Medical School Ann Arbor Michigan USA; ^4^ Department of Psychiatry University of Michigan Medical School Ann Arbor Michigan USA; ^5^ Department of Psychiatry Veterans Affairs Ann Arbor Healthcare System Ann Arbor Michigan USA; ^6^ Department of Anesthesiology University of Michigan Medical School Ann Arbor Michigan USA; ^7^ Department of Surgery, Section of Urology University of Chicago Pritzker School of Medicine Chicago Illinois USA

**Keywords:** abiraterone, anxiety, depression, enzalutamide, mental health, prostate neoplasms, quality of life

## Abstract

**Background:**

Abiraterone and enzalutamide are castration‐resistant prostate cancer (CRPC) therapies with potentially distinct associations with mental health symptoms given their differing antiandrogen targets.

**Methods:**

We used national Veterans Health Administration data to identify patients with CRPC who received first‐line abiraterone or enzalutamide from 2010 to 2017. Using Poisson regression, we compared outpatient mental health encounters per 100 patient‐months on drug between the abiraterone and enzalutamide cohorts adjusting for patient factors (e.g., age). We compared mental health encounters in the year before versus after starting therapy using the McNemar test.

**Results:**

We identified 2902 CRPC patients who received abiraterone (*n* = 1992) or enzalutamide (*n* = 910). We found no difference in outpatient mental health encounters between the two groups (adjusted incident rate ratio [aIRR] 1.04, 95% confidence interval [CI] 0.95–1.15). However, men with preexisting mental health diagnoses received 81.3% of the outpatient mental health encounters and had higher rates of these encounters with enzalutamide (aIRR 1.21, 95% CI 1.09–1.34). Among patients with ≥1 year of enrollment before and after starting abiraterone (*n* = 1139) or enzalutamide (*n* = 446), there was no difference in mental health care utilization before versus after starting treatment (17.0% of patients vs. 17.6%, *p* = 0.60, abiraterone; 16.4% vs. 18.4%, *p* = 0.26, enzalutamide).

**Conclusion:**

We found no overall differences in mental health care utilization between CRPC patients who received first‐line abiraterone versus enzalutamide. However, men with preexisting mental health diagnoses received the majority of mental health care and had more mental health visits with enzalutamide.

## Graphical abstract

In this retrospective cohort study of men with castration‐resistant prostate cancer who received their care in the Veterans Health Administration, there was no overall difference in mental health care utilization between those who received first‐line abiraterone versus enzalutamide. However, men with preexisting mental health diagnoses received the majority of mental health care and had more mental health visits with enzalutamide.

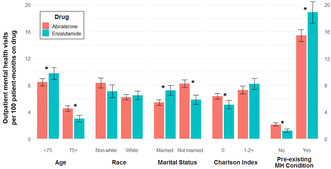

## 1 INTRODUCTION

Men with castration‐resistant prostate cancer (CRPC) have several treatment options with survival benefits.^1^, ^2^, ^3^, ^4^, ^5^, ^6^, ^7^, ^8^ Two treatments—abiraterone and enzalutamide—are oral agents that target the androgen axis and are widely used as first‐line therapies.^9^ Given their similar efficacy, patients and providers weigh the unique toxicity profiles of each drug, among other factors (e.g., cost), when choosing between the two. Abiraterone has a higher incidence of liver and cardiac toxicity and requires concurrent prednisone use whereas enzalutamide is associated with fatigue and falls and is contraindicated in those with a seizure disorder.^10^, ^11^, ^12^

To add to the distinctions between these two antiandrogens, post‐marketing data have conflicting findings as to whether abiraterone or enzalutamide is associated with an impact on emotional functioning.^13^, ^14^, ^15^, ^16^ Emotional functioning is of particular relevance to men with CRPC as the backbone treatment for the disease, androgen deprivation therapy (ADT), is associated with an increased risk of depression and anxiety.^17^, ^18^, ^19^ Abiraterone and enzalutamide are administered concurrently with ADT and further alter the androgen axis but in different ways. Abiraterone is a 17α‐hydroxylase inhibitor and further suppresses testosterone production whereas enzalutamide is an androgen receptor antagonist that crosses the blood–brain barrier but does not alter testosterone levels.^12^ Thus, the two drugs may have different impacts on emotional functioning. Two prospective studies conducted in “real world” practice suggest the possibility of worsened depression symptoms and emotional functioning with enzalutamide compared to abiraterone.^13^, ^14^ However, two other studies found no difference between the two drugs and subsequent depression, anxiety, and emotional functioning.^15^, ^16^ These studies enrolled a relatively small number of patients (100–200) who were followed for a limited period of time (2–12 months).

Given the conflicting data and that little is known about the longer‐term effects of abiraterone or enzalutamide on mental health outcomes, we sought to compare mental health care utilization between men who received abiraterone versus enzalutamide as first‐line therapy for CRPC in the Veterans Health Administration (VA). Veterans are twice as likely to be diagnosed with prostate cancer compared to the general population and are particularly vulnerable to the depression or anxiety that may arise from antiandrogen therapies due to their high baseline prevalence of mental health conditions.^20^ Over 33% of Vietnam era Veterans, the group that makes up the majority of Veterans with prostate cancer given their age, report symptoms of depression compared to 16% of age‐matched non‐Veterans.^21^ Understanding the impact of CRPC therapies on mental health is paramount as these men face multiple risk factors for mental health symptoms: their military service, advanced cancer diagnosis, ADT, and now potentially advanced antiandrogen therapies. Thus, we used a large national cohort of men with CRPC and the long‐term follow up in the VA electronic medical record to better understand the real‐world mental health implications of antiandrogen therapies in CRPC.^22^, ^23^

## 2 MATERIALS AND METHODS

### 2.1 Study cohort

**FIGURE 1:**
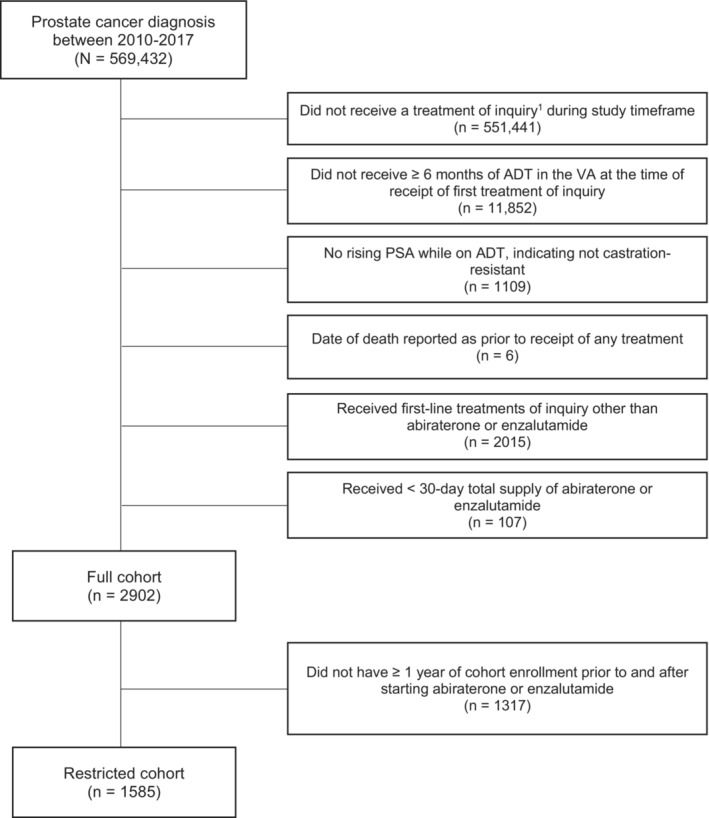
Development of analytic cohorts of men with CRPC who received first‐line abiraterone or enzalutamide. ^1^Abiraterone, cabazitaxel, docetaxel, enzalutamide, ketoconazole, mitoxantrone, radium‐223, sipuleucel‐T. ADT, androgen deprivation therapy; PSA, prostate specific antigen; VA, Veterans Health Administration.

Using the VA Corporate Data Warehouse (RRID:SCR_011566), consisting of medical record data from 130 health systems, we identified men with a diagnosis of prostate cancer using International Classification of Diseases, Ninth Revision (ICD‐9) code 185 for 2010–2015, and ICD‐10 code C61 for 2016–2017. Using pharmacy claims, we then identified men who received a treatment for CRPC (i.e., abiraterone, cabazitaxel, docetaxel, enzalutamide, ketoconazole, mitoxantrone, radium‐223, or sipuleucel‐T). Castration resistance was defined by requiring patients receive ADT for ≥6 months before starting a CRPC treatment and have a rising prostate specific antigen (PSA) while receiving ADT. ADT was defined as ≥2 injections of a gonadotropin‐releasing hormone analogue ≤8 months before receipt of a CRPC therapy or an orchiectomy in the prior 12 months. We then limited our cohort to those who received first‐line abiraterone or enzalutamide for ≥30 days (full cohort). Finally, for comparing mental health outcomes in the 1 year before and after starting abiraterone or enzalutamide, we used a restricted cohort of those with ≥1 year of cohort enrollment prior to and after starting abiraterone or enzalutamide (restricted cohort) in order to have a common time frame of comparison for our outcomes of interest. Figure 1 illustrates the development of our analytic cohorts.

### 2.2 Demographics & clinical characteristics

Patient age at initiation of abiraterone or enzalutamide, race, marital status, Charlson comorbidity index (CCI), rural vs. urban residence (based on patient zip code), treating facility complexity, preexisting mental health conditions, PSA at start of abiraterone or enzalutamide, presence of metastatic disease, and baseline opioid use were abstracted. The CCI was determined using ICD‐9 and ICD‐10 codes for relevant conditions in the 1 year prior to initiation of abiraterone or enzalutamide. Treating facility complexity was determined by the VA Support Service Center's facility complexity model.^24^ Preexisting mental health conditions (depression, anxiety, post‐traumatic stress disorder (PTSD), adjustment disorder, substance use disorder, bipolar disorder, schizophrenia, and attempted self‐harm) were identified using ICD‐9 and ICD‐10 codes (Supplemental Methods). Metastatic disease at the initiation of abiraterone or enzalutamide was determined by a validated, VA‐specific natural language processing tool.^24^ Opioid use was defined as having ≥30‐day prescription within 90 days of starting abiraterone or enzalutamide.

### 2.3 Outcomes

For our restricted cohort of patients who had ≥1 year of cohort enrollment before and after starting abiraterone or enzalutamide, we determined four outcomes: (1) the proportion of patients who received an incident mental health diagnosis, (2) the number of outpatient, emergency, or inpatient psychiatric encounters, (3) the proportion of patients with these encounters, and (4) psychotropic drug prescriptions ascertained using pharmacy claims. Psychiatric encounters were defined as encounters where the primary diagnosis was a mental health condition (depression, anxiety, PTSD, adjustment disorder, substance use disorder, bipolar disorder, schizophrenia, and attempted self‐harm) based on ICD‐9 and ICD‐10 codes. Polypharmacy was defined as having >1 psychotropic drug class prescription simultaneously for ≥90 days.

For our full cohort, we determined the number of outpatient psychiatric encounters. We counted one encounter per calendar day for a given patient to reduce potential duplicates.

### 2.4 Statistical analysis

Baseline demographic and clinical characteristics of the study cohort were compared using a *t*‐test when continuous and a chi‐square test when categorical.

For our restricted cohort, using the Wilcoxon signed rank test and McNemar test, we compared the following outcomes in the 1 year prior to initiation of abiraterone or enzalutamide with the 1 year after starting therapy: (1) the number of outpatient, emergency, and inpatient psychiatric encounters, (2) the proportion of patients who received this care, and (3) the proportion of patients who received psychotropic drug prescriptions.

For our full cohort, incidence rates (IR) of outpatient mental health encounters—defined as the number of outpatient mental health encounters during the time on therapy (first drug prescription fill to the end date of the last drug prescription fill) divided by the number of patient‐months on therapy to account for the varying duration of time on treatment—were calculated for the abiraterone and enzalutamide cohorts. The IRs in the abiraterone and enzalutamide cohorts were compared using the incidence rate ratio (IRR). An adjusted IRR was estimated using a Poisson regression model that adjusted for age, CCI, marital status, and presence of a preexisting mental health diagnosis, and included an offset equal to the natural logarithm of the time on treatment. Finally, the IRs, IRRs, and adjusted IRRs were stratified by age, race, marital status, CCI, and presence of a preexisting mental health diagnosis. The adjusted results for the stratified variable of interest excluded the given variable as a covariate (e.g., the adjusted IRR for CCI adjusted for age, marital status, and presence of a preexisting mental health diagnosis but not CCI).

Statistical testing was two‐sided with a level of significance set at *p* = 0.05. Analysis was performed using the R Project for Statistical Computing, v 4.1.2 (RRID:SCR_001905). This study was approved by the VA Ann Arbor Healthcare System Internal Review Board.

## 3 RESULTS

### 3.1 Sample characteristics

Among 569,432 Veterans identified with prostate cancer between 2010 and 2017, 1992 received abiraterone and 910 enzalutamide as first‐line treatment for CRPC (Figure 1).

### 3.2 Cohort characteristics

Men who received enzalutamide were older, had more comorbid conditions, and had a lower PSA at start of treatment (all *p* < 0.01) than those who received abiraterone (Table 1). More men who received abiraterone had baseline opioid use than those who received enzalutamide (35.9% vs. 30.0%, *p* < 0.01). There was no difference in race, treating facility complexity, presence of metastatic disease, or prevalence of preexisting mental health diagnoses between the two groups; 33.3% of men who received abiraterone and 29.7% who received enzalutamide had a preexisting mental health condition. Depression was most common, followed by substance use disorder and PTSD/adjustment disorder in both cohorts.

**TABLE 1:** Patient characteristics.

	Abiraterone *N* = 1992 (*n*, %)		Enzalutamide *N* = 910 (*n*, %)		*p*‐value^a^
Age, years (mean, range)	74.4	(51–100)	75.5	(53–100)	0.003
Race					0.11
White	1329	(66.7)	569	(62.5)	
Black	540	(27.1)	283	(31.1)	
Other	28	(1.4)	17	(1.9)	
Unknown	95	(4.8)	41	(4.5)	
Marital status					0.91
Married	1046	(52.5)	480	(52.7)	
Not married	946	(47.5)	430	(47.3)	
Service Era					
World War II	260	(13.1)	100	(11.0)	0.12
Korea	453	(22.7)	206	(22.6)	0.95
Vietnam	1156	(58.0)	545	(59.9)	0.35
Gulf	28	(1.4)	17	(1.9)	0.35
Other	204	(10.2)	94	(10.3)	0.94
Charlson Comorbidity Index					<0.001
0	1122	(56.3)	451	(49.6)	
1	451	(22.6)	201	(22.1)	
2+	419	(21.0)	258	(28.4)	
Rural vs. urban home zip code					0.11
Highly Rural	26	(1.3)	21	(2.3)	
Rural	637	(32.0)	303	(33.3)	
Urban	1326	(66.6)	586	(64.4)	
Missing	3	(0.2)	0	(0)	
Treating facility complexity					0.68
1a–High	936	(47.0)	399	(43.8)	
1b–High	509	(25.6)	247	(27.1)	
1c–High	336	(16.9)	160	(17.6)	
2–Medium	115	(5.8)	55	(6.0)	
3–Low	95	(4.8)	49	(5.4)	
PSA at start of abiraterone or enzalutamide (median, IQR)	38.5	(14.2–115)	29.1	(11.6–85.6)	0.008
Metastatic disease at start of abiraterone or enzalutamide	1655	(83.1)	759	(83.4)	0.83
Opioid use^b^	715	(35.9)	273	(30.0)	0.002
Preexisting mental health conditions					
Any	664	(33.3)	270	(29.7)	0.18
Depression	309	(15.5)	140	(15.4)	0.93
Anxiety	132	(6.6)	59	(6.5)	0.88
PTSD/adjustment disorder	220	(11.0)	94	(10.3)	0.57
Substance use disorder	288	(14.5)	115	(12.6)	0.18
Bipolar disorder	26	(1.3)	8	(0.9)	0.32
Schizophrenia	31	(1.6)	14	(1.5)	0.97
Attempted self‐harm	0	(0.0)	1	(0.1)	0.14
>1 diagnosis	245	(12.3)	110	(12.1)	0.76

Abbreviations: IQR, interquartile range; PSA, prostate specific antigen; PTSD, posttraumatic stress disorder.

^a^ *p*‐values calculated from a *t*‐test (continuous) or chi‐square test (categorical). A single *p*‐value is reported for categorical variables in which categories are mutually exclusive.

^b^ Receipt of ≥30‐day prescription within 90 days of starting abiraterone or enzalutamide.

### 3.3 Mental health diagnoses & care utilization before versus after starting abiraterone or enzalutamide

After restricting patients to those who had ≥1 year of cohort enrollment before and after initiation of abiraterone or enzalutamide, our restricted cohort consisted of 1585 men (1139 received abiraterone and 446 enzalutamide). Between 12%–14% of patients received an incident mental health diagnosis after starting abiraterone or enzalutamide (Table 2). Between 16%–18% of patients were engaged in mental health care in both the abiraterone and enzalutamide groups with no significant difference before versus after starting therapy (*p* = 0.60, 0.26, respectively). Significantly more patients who received abiraterone were prescribed a psychotropic drug after starting abiraterone than before (33.1% vs. 30.5%, *p* = 0.01). There was no difference in the proportion of patients who received a psychotropic drug before versus after starting enzalutamide.

**TABLE 2:** Mental health diagnoses and care utilization before versus after starting first‐line abiraterone or enzalutamide for CRPC.

	Abiraterone *N* = 1139					Enzalutamide *N* = 446				
	Pre‐^a^		Post‐^b^		*p*‐value^c^	Pre‐^a^		Post‐^b^		*p*‐value^c^
	*n*	(%)	*n*	(%)		*n*	(%)	*n*	(%)	
Incident mental health diagnosis										
Any			165	(14.4)	N/A			56	(12.6)	N/A
Depression			74	(6.5)				24	(5.4)	
Anxiety			39	(3.4)				12	(2.7)	
PTSD/adjustment disorder			50	(4.4)				24	(5.4)	
Substance use disorder			40	(3.5)				8	(1.8)	
Bipolar disorder			3	(0.3)				1	(0.2)	
Schizophrenia			2	(0.2)				0	(0.0)	
Attempted self‐harm			2	(0.2)				0	(0.0)	
>1 diagnosis			43	(3.8)				13	(2.9)	
Outpatient psychiatric care										
Events	1139		1038		0.62	599		483		0.50
Patients engaged	194	(17.0)	201	(17.6)	0.60	73	(16.4)	82	(18.4)	0.26
Emergency psychiatric care										
Events	5		13		0.11	12		3		0.82
Patients engaged	5	(0.4)	11	(1.0)	0.21	3	(0.7)	3	(0.7)	>0.99
Inpatient psychiatric hospitalizations										
Events	4		5		0.78	1		1		>0.99
Patients engaged	4	(0.4)	5	(0.4)	>0.99	1	(0.2)	1	(0.2)	>0.99
Psychiatric drug use										
Total	347	(30.5)	377	(33.1)	0.01	119	(26.7)	130	(29.1)	0.21
SSRIs	156	(13.7)	169	(14.8)	0.10	63	(14.1)	65	(14.6)	0.83
SNRIs	60	(5.3)	54	(4.7)	0.39	17	(3.8)	25	(5.6)	0.10
Atypical antidepressants^d^	75	(6.6)	73	(6.4)	0.90	22	(4.9)	30	(6.7)	0.14
Benzodiazepines	113	(9.9)	129	(11.3)	0.10	26	(5.8)	24	(5.4)	0.82
Antipsychotics	30	(2.6)	31	(2.7)	>0.99	14	(3.1)	18	(4.0)	0.34
Tricyclic antidepressants	31	(2.7)	26	(2.3)	0.38	11	(2.5)	5	(1.1)	0.08
MAO‐Is	0	(0.0)	0	(0.0)	N/A	1	(0.2)	0	(0.0)	N/A
Buspirone	12	(1.1)	17	(1.5)	0.23	5	(1.1)	7	(1.6)	0.62
Lithium	4	(0.4)	3	(0.3)	>0.99	0	(0.0)	0	(0.0)	N/A
Substance use therapies^e^	9	(0.8)	13	(1.1)	0.39	4	(0.9)	7	(1.6)	0.37
Polypharmacy^f^	94	(8.3)	95	(8.3)	>0.99	30	(6.7)	38	(8.5)	0.10

Abbreviations: MAO‐I, monoamine oxidase inhibitor; PTSD, posttraumatic stress disorder; SNRI, serotonin norepinephrine reuptake inhibitor; SSRI, selective serotonin reuptake inhibitor.

^a^ Limited to 12 months prior to start of drug.

^b^ Limited to 12 months after start of drug.

^c^ Wilcoxon signed rank test when comparing number of events; McNemar test when comparing proportion of patients.

^d^ Atypical antidepressants = bupropion, mirtazapine.

^e^ Substance use therapies = acamprosate, buprenorphine, disulfiram, methadone, naltrexone.

^f^ Receipt of >1 psychiatric drug class simultaneously for ≥90 days.

### 3.4 Mental health care utilization in abiraterone versus enzalutamide cohorts

**FIGURE 2:**
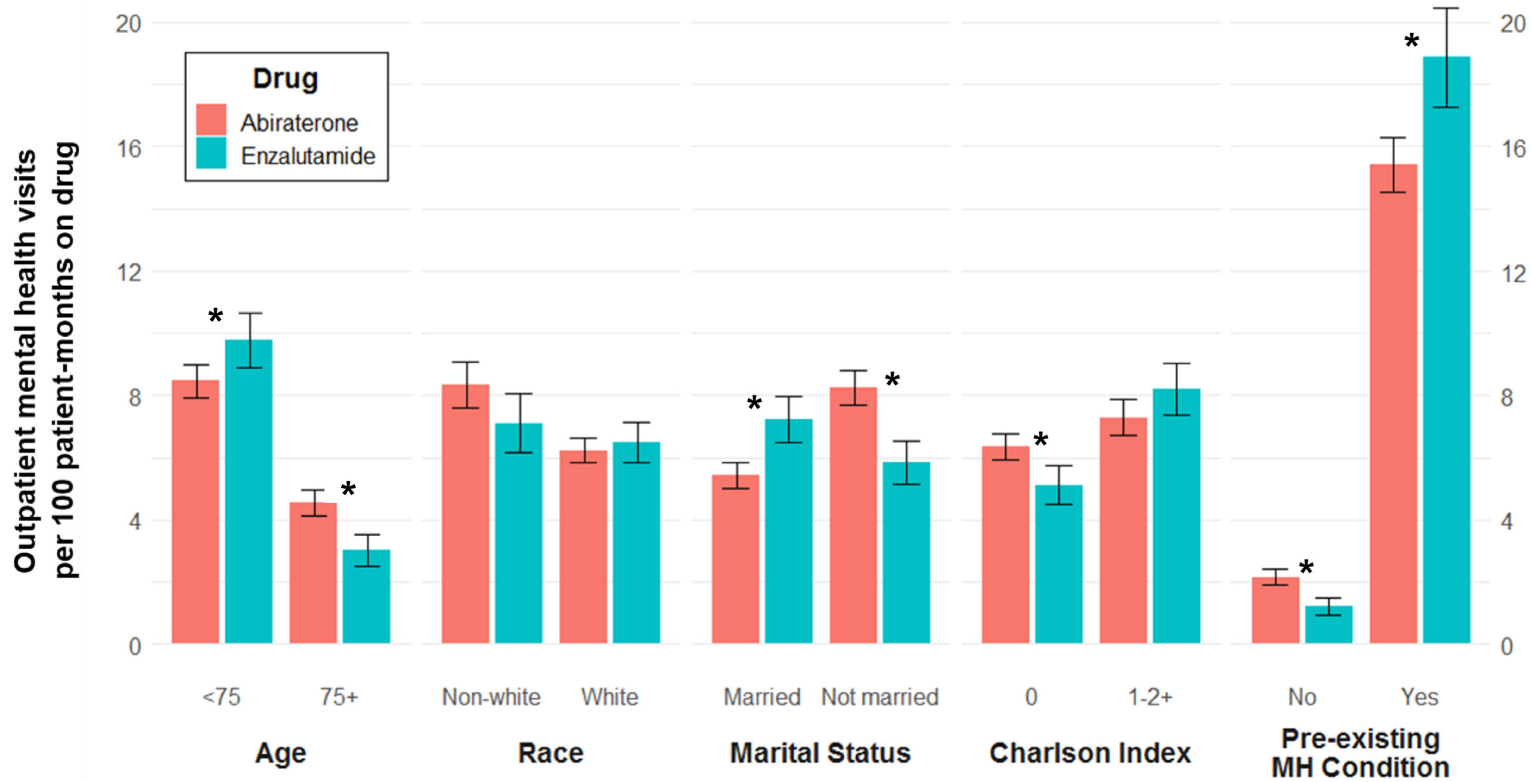
Outpatient mental health visits in men with CRPC who received first‐line abiraterone versus first‐line enzalutamide. * Reflects unadjusted incident rate ratios for which the 95% confidence interval does not include 1.00. Errors bars reflect 95% confidence intervals. MH, mental health.

In our full cohort, we found no difference in the number of outpatient mental health encounters per 100 patient‐months on drug between the abiraterone and enzalutamide groups (Table 3). Men with preexisting mental health conditions accounted for 81.3% of the mental health encounters. In a subgroup analysis, men who were younger (<75 years old), married, had more comorbidities, or had a preexisting mental health diagnosis had more mental health encounters with enzalutamide. Men who were older (≥75 years old), not married, or did not have a preexisting mental health condition had more mental health encounters with abiraterone. The number of mental health visits per 100 patient‐months on drug are demonstrated in Figure 2. Men with preexisting mental health conditions receiving enzalutamide had 18.9 mental health visits per 100 patient‐months on drug, 3.5 more than those receiving abiraterone. In comparison, men without a preexisting mental health condition receiving enzalutamide had 1.2 mental health visits per 100 patient‐months on drug and those receiving abiraterone 2.2.

**TABLE 3:** Outpatient mental health visits in men with CRPC who received first‐line abiraterone versus first‐line enzalutamide.

	Abiraterone [A]				Enzalutamide [E]				Unadjusted IRR [E]/[A], (95% CI)	Adjusted IRR [E]/[A], (95% CI)
	Total patients	Total MH visits	Patient‐months on drug (PM)	MH visits per 100 PM	Total patients	Total MH visits	Patient‐months on drug (PM)	MH visits per 100 PM		
Overall	1992	1501	22,307	6.73	910	630	9571	6.58	0.98 (0.89, 1.07)	1.04 (0.95, 1.15)
Age										
<75	1042	1049	12,402	8.46	444	493	5045	9.77	1.16 (1.04, 1.28)	1.23 (1.10, 1.37)
≥75	950	452	9905	4.56	466	137	4526	3.03	0.66 (0.55, 0.80)	0.69 (0.57, 0.83)
Race										
White	1329	937	15,040	6.23	569	389	6006	6.48	1.04 (0.92, 1.17)	1.08 (0.95, 1.21)
Not white	539	475	5688	8.35	284	207	2909	7.12	0.85 (0.72, 1.00)	0.89 (0.75, 1.04)
Marital status										
Married	1046	653	12,037	5.42	480	365	5035	7.25	1.34 (1.17, 1.52)	1.40 (1.23, 1.59)
Not married	946	848	10,270	8.26	430	265	4536	5.84	0.71 (0.62, 0.81)	0.77 (0.67, 0.89)
Charlson Comorbidity Index										
0	1122	855	13,457	6.35	451	257	5021	5.12	0.81 (0.70, 0.92)	0.94 (0.82, 1.08)
1+	870	646	8850	7.30	459	373	4550	8.20	1.12 (0.99, 1.27)	1.19 (1.05, 1.35)
Preexisting MH diagnosis										
Yes	664	1185	7681	15.43	270	548	2904	18.87	1.22 (1.10, 1.35)	1.21 (1.09, 1.34)
No	1328	316	14,626	2.16	640	82	6667	1.23	0.57 (0.44, 0.72)	0.55 (0.43, 0.69)

*Note*: Adjusted IRRs were estimated using Poisson regression models that adjusted for continuous age, number of Charlson comorbidities, marital status, and whether there was a pre‐existing mental health diagnosis. The adjusted results for the stratified variable of interest excluded its corresponding adjustment variable, where applicable (e.g. the adjusted IRRs for stratified analysis of categorical age adjusted for Charlson comorbidities, marital status, and preexisting mental health diagnosis but did not adjust for continuous age).

Abbreviations: CI, confidence interval; CRPC, castration‐resistant prostate cancer; IRR: incidence rate ratio; MH, mental health; PM, patient‐months on drug.

## 4 DISCUSSION

In a nationwide cohort of 2902 men with CRPC in which nearly one‐third had a preexisting mental health condition, we found over 12% of patients developed a new mental health diagnosis after starting abiraterone or enzalutamide, resulting in over 40% of our cohort having a mental health diagnosis. We found no overall difference in mental health care utilization with starting first‐line abiraterone or enzalutamide. In comparing outcomes between men who received abiraterone versus enzalutamide, there was no difference in the rate of outpatient mental health encounters. In a subgroup analysis, men <75 years old, who were married, had more comorbidities, or had preexisting mental health conditions had more mental health encounters with enzalutamide while older men, unmarried men, and those without preexisting mental health conditions had more mental health encounters with abiraterone. Men with preexisting mental health conditions accounted for the majority of outpatient mental health encounters. They also had the largest absolute difference in mental health encounters favoring abiraterone over enzalutamide out of all subgroups analyzed.

Prior literature contains conflicting data regarding mental health outcomes experienced by men with CRPC receiving abiraterone versus enzalutamide. Our findings are consistent with two smaller prospective studies that demonstrated no difference in emotional functioning, depression or anxiety as measured by patient surveys between men who received abiraterone versus enzalutamide.^15^, ^16^ Our study expands on this work by demonstrating similar results using an alternate outcome measurement (i.e., mental health care utilization) which captures formally diagnosed mental health conditions in a much larger nationwide sample with patients treated over 7 years.

However, our results are contrary to a randomized phase II study that found more men who received enzalutamide reported depression symptoms than those who received abiraterone during weeks 4–12 of treatment.^13^ Another prospective study that followed patients for 12 months demonstrated less clinically meaningful worsening of emotional functioning with abiraterone compared with enzalutamide.^14^ Our work suggests that, while overall there may not be a difference in mental health care utilization between men receiving abiraterone versus enzalutamide, certain subgroups may be prone to worsening mental health symptoms with one drug versus the other. Notably, the literature has not examined the role of comorbidities, preexisting mental health conditions, or marital status on mental health outcomes in men receiving abiraterone or enzalutamide. Nearly one‐third of our cohort had a preexisting mental health condition. We found these men accounted for the majority of the cohort's mental health visits with a more substantial absolute difference in mental health visits favoring abiraterone over enzalutamide. Thus, our findings raise the possibility that men with preexisting mental health conditions are particularly at risk for increased need for mental health care with enzalutamide compared to abiraterone, perhaps due to enzalutamide's direct central nervous system effects.^12^ On the other hand, we found certain subgroups had more mental health encounters with abiraterone, which was unexpected given the literature has thus far only suggested either no difference or worsening of emotional functioning with enzalutamide. We hypothesize that the higher monitoring burden (blood work every other week for 3 months, the need to closely monitor blood pressure) and the development of cardiac and metabolic diseases more often seen with abiraterone may lend to increased distress for older men and for those who are not married with marital status being potentially reflective of having a social support structure.^10^ Finally, we noted men <75 years old had more mental health visits overall compared to men ≥75 years old. This is likely due to most men <75 years old being Vietnam era Veterans who have a higher prevalence of mental health conditions compared to World War II and Korean War era Veterans.^25^

Our study does have limitations. First, our outcome was mental health encounters as opposed to direct measurement of symptoms via patient surveys or provider interview. Thus, our outcome is limited to individuals who both experience symptoms and sought care and would exclude those who discontinued abiraterone or enzalutamide due to mental health symptoms without seeking care. However, we broadly defined mental health care as any encounter with a primary mental health diagnosis which would capture symptoms addressed by any primary or cancer care provider as opposed to only visits with a psychologist or psychiatrist. In addition, while mental health care access has substantial barriers in the private sector, the VA has a dedicated focus on routine screening for mental health conditions and mental health care access given the high prevalence and service‐related nature of mental health conditions in Veterans. Thus, Veterans may experience lower barriers in terms of appointment access and cost to mental health care in the VA.^23^ Using mental health encounters as an outcome likely encompasses many of the patients who experience mental health symptoms. Second, given the retrospective, non‐randomized nature of our study, there are possibly unmeasured factors associated with the receipt of abiraterone or enzalutamide that may impact mental health care utilization. However, we ensured robust capture of the known relevant and available variables (i.e., age, race, comorbidities, preexisting mental health conditions, treating facility complexity).

Our findings suggest that overall, there is no difference in mental health care utilization among men with CRPC who receive first‐line abiraterone versus enzalutamide. They also indicate the greatest mental health care utilization occurred among men with preexisting mental health conditions. Men with more comorbidities or a preexisting mental health condition experienced more mental health visits with enzalutamide whereas older men and unmarried men experienced more mental health visits with abiraterone. These results warrant further investigation in larger, prospective studies. If substantiated, these findings may be important in shaping treatment choice in CRPC and informing the critical patient‐family‐provider discussions regarding the risks and benefits of therapy. Furthermore, abiraterone and enzalutamide are both now standard of care for metastatic hormone‐sensitive prostate cancer (mHSPC) where men are exposed to these drugs continuously and indefinitely for upwards of 4–5 years.^11^, ^26^, ^27^, ^29^ Abiraterone is also approved for 2 years of use in men with locally advanced prostate cancer.^28^ Enzalutamide has been recently studied in low‐risk and intermediate‐risk localized prostate cancer which affects over 30,000 men annually in the United States.^30^, ^31^ As abiraterone and enzalutamide use expands to tens of thousands of men, a complete understanding of the mental health implications of these agents is even more critical in order to ensure a true informed decision is made by patients and to develop effective interventions in order to improve treatment adherence and quality of life.

## 5 CONCLUSION

Our findings suggest that men with CRPC who receive first‐line abiraterone or enzalutamide do not experience an increase in mental health care utilization after starting therapy. In addition, there is no difference in mental health care utilization between those who receive abiraterone versus enzalutamide. However, men with preexisting mental health conditions who accounted for the majority of mental health care delivered experienced more mental health visits with enzalutamide compared to abiraterone. Further work should investigate the incidence of depression and anxiety with antiandrogen therapies in mHSPC where these drugs will be used for years, the factors associated with the development of depression and anxiety and subsequently, the development of interventions to optimize the quality of life of men with advanced prostate cancer.

## AUTHOR CONTRIBUTIONS

**Phoebe A. Tsao:** Conceptualization (equal); methodology (equal); validation (equal); writing – original draft (equal); writing – review and editing (equal). **Jennifer Burns:** Data curation (equal); formal analysis (equal); methodology (equal); validation (equal); writing – review and editing (equal). **Kyle Kumbier:** Formal analysis (equal); methodology (equal); validation (equal); writing – review and editing (equal). **Jordan B. Sparks:** Project administration (equal); writing – review and editing (equal). **Shami Entenman:** Methodology (equal); validation (equal); writing – review and editing (equal). **Lindsey Bloor:** Methodology (equal); validation (equal); writing – review and editing (equal). **Amy Bohnert:** Methodology (equal); validation (equal); writing – review and editing (equal). **Ted Skolarus:** Methodology (equal); validation (equal); writing – review and editing (equal). **Megan Elizabeth Veresh Caram:** Conceptualization (equal); funding acquisition (equal); methodology (equal); supervision (equal); validation (equal); writing – review and editing (equal).

## FUNDING INFORMATION

This work was supported by a Prostate Cancer Foundation Young Investigator Award (MEVC). PAT is supported by the Prostate Cancer Foundation's Precision Oncology Program for Cancer of the Prostate. TAS and MEVC are supported by National Cancer Institute grants R37CA222885 and R01CA242559. ASBB is supported by RO1MH131617, VAI01HX003411, VAC19‐21‐278, Blue Cross Blue Shield, and the State of Michigan.

## CONFLICT OF INTEREST STATEMENT

None.

## ETHICS STATEMENT

This study was approved by the VA Ann Arbor Healthcare System Internal Review Board.

## PATIENT CONSENT

Patient consent was not required for this study. Data were derived from de‐identified patient records housed in the VA Corporate Data Warehouse.

## Supporting information


Data S1.

## Data Availability

The statistical code that supports the findings of this study is available from the corresponding author (PAT) upon request. The raw data is not available due to its sensitive nature and ownership by the Veterans Health Administration.
