# Supplementary material for: Mental health care utilization among men with castration‐resistant prostate cancer receiving abiraterone or enzalutamide
Source: Cancer Med. 2023 Jun 16;12(15):16490–501. doi: 10.1002/cam4.6237 (PMC10469813; doi:10.1002/cam4.6237)
Supplement: Supplementary file 1 — Data S1. [file CAM4-12-16490-s001.docx]

**Supplemental Methods. Mental health conditions definitions.**

| **Diagnosis** | **Codes** |
| --- | --- |
| Depression | ICD-9: 296.2, 296.3, 296.5, 296.6, 298.0, 301.10, 301.12, 301.13, 309.0, 309.1, 311  ICD-10: F32.x, F33.x, F34.x, F38.x, F39 |
| Anxiety | ICD-9: 298.84, 300.0, 300.01, 300.02, 300.09, 308  ICD-10: F41.x, F42.x |
| PTSD/adjustment disorder | ICD-9: 309.x  ICD-10: F43, F43.0, F43.1, F43.2, F43.8, F43.9 |
| Substance use disorder | ICD-9: 291.x, 303, 304.x, 305.x  ICD-10: F10.x, F11.x, F12.x, F13.x, F14.x, F15.x, F16.x, F18.x, F19.x |
| Bipolar disorder | ICD-9: 296.0, 296.4, 296.5, 296.6, 296.7, 296.8  ICD-10: F30.x, F31.x |
| Schizophrenia | ICD-9: 295.x  ICD-10: F20.x, F21, F25.x, F28, F29 |
| Attempted self-harm | ICD-9: E950.x, E951.x, E952.x, E953.x, E954.x, E955.x, E956.x, E957.x, E958.x, E959.x  ICD-10: X71.x, X72.x, X73.x, X74.x, X75.x, X76.x, X77.x, X78.x, X79.x, X80.x, X81.x, X82.x, X83.x, T14.91 |
| Abbreviations: ICD-9, International Classification of Diseases, Ninth Revision; ICD-10, International Classification of Diseases, Tenth Revision | |
